# The historical decline of tobacco smoking among United States physicians: 1949–1984

**DOI:** 10.1186/1617-9625-4-9

**Published:** 2008-09-09

**Authors:** Derek R Smith

**Affiliations:** 1 WorkCover New South Wales Research Centre of Excellence, School of Health Sciences, Faculty of Health, University of Newcastle, Ourimbah, Australia

## Abstract

**Background:**

Tobacco use became an ingrained habit in the United States (US) following the First World War and a large proportion of physicians, similar to the general population, were smokers. The period from 1949 to 1984 was a pivotal era of change however, as the medical profession, like the society it served, became increasingly aware of the dangers that tobacco incurred for health.

**Methods:**

An extensive review targeted all manuscripts published in academic journals between 1949 and 1984 that related to tobacco smoking among US physicians. The study was undertaken in 2007–08 with an internet search of relevant medical databases, after which time the reference lists of manuscripts were also examined to find additional articles.

**Results:**

A total of 57 manuscripts met the inclusion criteria. From a research perspective, the methodology and coverage of smoking surveys ranged from detailed national investigations, to local medical association surveys, and journal readership questionnaires. From a historical perspective, it can be seen that by the 1950s many US physicians had begun questioning the safety of tobacco products, and by the 1960s and 1970s, this had resulted in a continuous decline in tobacco use. By the 1980s, few US physicians were still smoking, and many of their younger demographic had probably never smoked at all.

**Conclusion:**

Although the quality and coverage of historical surveys varied over time, a review of their main results indicates a clear and consistent decline in tobacco use among US physicians between 1949 and 1984. Much can be learned from this pivotal era of public health, where the importance of scientific knowledge, professional leadership and social responsibility helped set positive examples in the fight against tobacco.

## Background

Tobacco use became an ingrained habit in the Unites States (US) following the First World War [1], with per capita tobacco consumption increasing from six pounds in the 1880s, to approximately 13 pounds per person in the mid 20^th ^century [2]. By this time cigarette smoking was the norm, and a large proportion of American physicians, similar to the general population, were smokers. Increasing public anxiety led to various advertising campaigns referring directly to physicians, in an attempt to assure consumers that tobacco products were safe [3,4]. Various American medical journals also carried tobacco advertisements during this period [5], although such practices were not limited to the US [6,7]. By the mid 1950s however, amidst growing public concern, tobacco industry strategists had determined that physicians were no longer credible in cigarette advertising, and commercials incorporating doctors began to slowly disappear [4]. Aside from increasing awareness within the medical community and their removal from advertising campaigns, many changes in the national smoking demographic were also catalysed by the release of the Surgeon General's landmark report in 1964 [8], where it was unequivocally decided that smoking was a health hazard of sufficient importance in the United States to warrant remedial action. The role of the American medical profession in meeting this challenge was therefore, abundantly clear.

Although all health professionals can contribute to tobacco control [9], physicians have always had an important responsibility to convince their patients not to smoke [10,11]. Physicians are generally viewed as exemplars by the community, and as such, their office and hospital should be a model of non-smoking behaviour [12]. They also serve as providers of support, information and encouragement in helping patients to achieve such a goal [13]. For these reasons and more, it is essential that physicians themselves do not smoke. It has been previously noted that the medical profession tends to give up smoking earlier than the general population, as the dangers become clear [14] and physicians are in a good position to recognise the significance of scientific findings. While smoking in the medical profession has generally declined worldwide since the 1950s [15], tobacco control measures have not been uniformly successful, and physicians in some countries still consume tobacco at relatively high rates [16]. On the other hand, major gains have now been achieved in the United States; most contemporary physicians in this country do not currently smoke, and most younger ones probably never have [16].

How this situation came to be reveals an important period in the history of tobacco control, as physicians progressed from being cigarette smokers themselves, to quitters and then to anti-smoking role models. Although many investigations have looked at the progression of smoking in the general American population, few historical studies have specifically reviewed the changing nature of smoking habits among US doctors. Given that physicians have been largely absent from direct tobacco-related advertising since the 1950s and that the prevalence of smoking among US adults has almost halved since the 1960s [17], it can be assumed that this time period represents the beginning of a major change within American society. Indeed, it was noted that between 1952 and 1957, marked changes were already occurring in the smoking habits of many American doctors [18]. By 1971, physicians were being advised to "heal thyself" [19]. On the other hand, a recent review of smoking in the medical profession [16] has already elucidated the contemporary prevalence of smoking among physicians in the US and elsewhere. For these reasons, the current study was conducted as a comprehensive review of US physicians' smoking habits during the key transitional period in tobacco usage habits, from post World War Two until the early 1980s. Due to a lack of published material before that time, it was considered impractical to extend the study any further back than 1949, the year in which the first study appears to have been conducted [20]. By 1984, it had been noted that almost all US physicians had stopped smoking [21], and hence, this point was used as the termination year.

## Methods

This study was conducted as an extensive literature review of all manuscripts relating to tobacco smoking among United States physicians that had been published in peer-reviewed journals between 1949 and 1984. Unpublished articles were not considered, and for consistency, only English-language reports were included. The literature review was performed in 2007–08 with an internet search of the National Library of Medicine's *Medline*^® ^database incorporating all years between 1949 and 1984. The Medical Subject Headings (MeSH) of 'smoking', 'tobacco', 'physician' and 'United States' were initially used, followed by the use of additional keyword variations such as 'smoke', 'doctor', 'America', and so on. The reference lists of all manuscripts located using these initial search criteria were then examined to find additional publications that may not have been listed on modern search engines. A large proportion of manuscripts were eventually located using the latter method. Manuscripts were initially arranged by location (single states, multiple states, national surveys, journal-based surveys and those performed in unspecified locations), and then in descending order, depending on the year in which the survey had been undertaken. All articles were assigned a reference number based on the abovementioned criteria. As the results of some investigations were published over more than one journal article, some studies have two to three corresponding references. Investigations that had clearly divided their data into different groups, either by location or by year of study, were separated into multiple sections.

All data was placed in a table and arranged as follows: author of study, year of publication and reference number as used in this manuscript (author codes: ACS = American Cancer Society, CA:ACJC = CA: A Cancer Journal for Clinicians, CMA = California Medical Association, NCSH = National Clearinghouse for Smoking and Health), year in which the study was conducted (in cases where the study year was not listed, the publication year is listed and marked with an asterisk), state in which the study was undertaken (AL = Alabama, CA = California, DL = Delaware, IL = Illinois, MS = Massachusetts, ML = Maryland, NB = Nebraska, NY = New York, PN = Pennsylvania, RI = Rhode Island, WI = Wisconsin), from where the participants were sourced (using terms as described in the manuscript) and whether their medical specialty was listed (if so, GP = General Practitioners, IM = Internal Medicine, O = Other or undefined speciality, OBG = Obstetrics/Gynaecology, PED = Paediatricians, PH/DN = Physicians/Dentists, PH/SG = Physicians/Surgeons, PUL = Pulmonary Physicians, PSY = Psychiatrists, RES = Medical Residents). Smoking status was defined as per the original manuscript and categorized (when available) with all smoking rates rounded to the nearest whole number and listed by gender, where possible (M = Male, F = Female, WM = White Male). If the type of tobacco smoked by physicians was not clearly defined, or if the smoker was a current smoker of pipes or cigars or both, this was indicated on the table. As some manuscripts listed only raw data or figures, certain smoking rates were calculated by the author and indicated as such on the table.

Survey response rates were rounded to the nearest whole number, and where multiple methods were used to follow-up initial non-responders (mainly telephone calls), this was indicated on the table. Unlisted response rates were calculated by the author, where possible. For physician's data that had been sourced from national surveys, the overall response rate of the entire survey is listed, and if the exact number of participating physicians was not listed, then the total number of participants in the study was indicated. National survey study codes were as follows: ACS = American Cancer Society, HIS = Health Interview Survey, NHIS = National Health Interview Survey and NORCS = National Opinion Research Center Survey. If the response rate of the survey was not specified and could not be manually calculated, this was indicated on the table. Additional information from the study (including smoking rates by medical speciality, where available), were also displayed.

## Results

An extensive literature search eventually located 57 studies (published in 53 journal articles) which met the inclusion criteria, as shown in Table S1 in Additional file 1. By location, the most commonly researched state was Rhode Island, where at least six different investigations of this topic appear to have been undertaken. Four studies (described in five manuscripts) had been undertaken in Massachusetts, four in California, two each in Connecticut, Florida, Oregon and Pennsylvania; with single investigations having been performed in Indiana and Wisconsin. At least eight studies had targeted physicians from multiple-states, with twelve national surveys, six conducted by medical journals, three conducted at conferences and one with an unspecified location. Most of the single state investigations had been conducted as postal surveys, although one had been "distributed" to physicians at a university health sciences centre [22], while another had "polled" members at county medical meetings [23]. At least eight studies that were predominately conducted as postal surveys had also used other methods, mainly telephone calls, to contact non-responders. Three studies had been conducted across multiple states, with a fourth investigation that followed the same group of physicians from four different states over a 20-year period. Of the twelve studies that involved a national sample of physicians, seven had sourced their data from larger, population-based surveys such as the National Health Interview Survey. Six investigations had been conducted among journal readers, presumably nationwide, with a further three studies performed at conferences or meetings and the location of one other investigation being unclear.

Sample sizes ranged from 45 to 56004, with state medical associations appearing to have had some of largest returns. Overall response rates ranged from 40% to 90% among the single state surveys, although for the majority of multiple state and national investigations, a response rate was not listed by the authors. The determination of absolute tobacco smoking rates was somewhat complicated, due to a lack of standardisation regarding the definition of 'current smoker' and a lack of consensus on exactly what product they smoked. Although most authors referred to their subjects as being either current smokers or non-smokers, the type of tobacco they smoked was classified in many different categories such as cigarettes only, pipes only, cigars only, pipes and cigars, pipes or cigars, cigarettes or pipes or cigars; and so on. This lack of standardization in tobacco smoking research has previously been noted among studies conducted with physicians [16], dentists [24] and nurses [25]; and probably arises due to the inherent difficulties in assessing smoking habits over time, and the fact that most investigations simply report the point-prevalence of smoking within a particular group.

**Figure 1:**
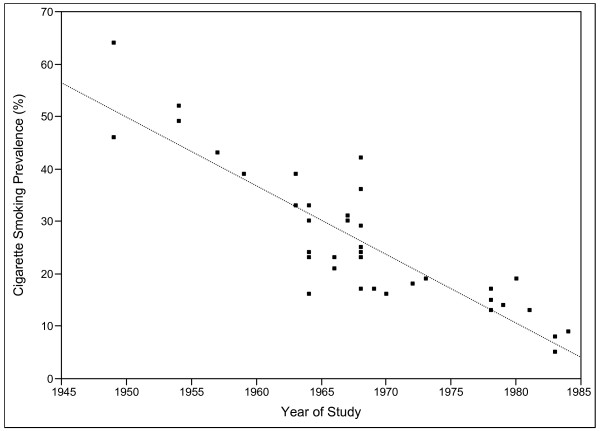
Decreasing Trend of Cigarette Smoking Prevalence among Physicians in the United States between 1949 and 1984.

Despite this fact, a large proportion of studies had listed the cigarette smoking prevalence rate among the physicians they surveyed, and much can be learned from the data. Firstly, from a summary of the retrieved manuscripts, it can be seen that absolute smoking prevalence rates declined from around 40% in the 1960s to less than 10% by the 1980s. Refer to Figure 1. A longitudinal study of one specific group of physicians over 20 years [20] revealed a major decline in cigarette smoking among them, with the rate falling from 64% to 30%. This situation was particularly evident in Rhode Island, where multiple smoking surveys have been conducted among physicians for a long period, and the group had almost become smoke-free by the early 1990s [26]. Other useful information on US physicians smoking habits and their associated trends was evident among studies that had utilised data from national population surveys, such as the American Cancer Society, as well as the National Health Interview Survey and the National Opinion Research Center Surveys.

## Discussion

### Societal Trends

On January 11, 1964, the Surgeon General's Advisory Committee on Smoking and Health released a groundbreaking report [8] where, after decades of debate, it was finally concluded that "Cigarette smoking is a health hazard of sufficient importance in the United States to warrant appropriate remedial action" (p.33). Prior to this time however, tobacco smoking had already become a regular and ingrained habit for most people by the turn of the 19^th ^century [1]. So well-accepted was the practice, that in 1922 Brill [27] had actually commented that "..one is more justified in looking with suspicion on the abstainer.." (p.444). Physicians were not immune to social forces, and evidently, a large proportion of them smoked tobacco products during this period. While the 1964 Surgeon General's Report was clearly a watershed for tobacco control advocates, a 1956 study group had earlier been tasked with evaluating all significant experiments, investigations and reports, relating to the topic of smoking and health [28]. In their report, published in 1957 [29], it was concluded that the smoking of tobacco products, particularly cigarettes, was an important health hazard. Similarly, the groundbreaking reports by Hammond and Horn in 1954 [30] and 1958 [31,32], had also done much to unsettle the American public's confidence in the safety of tobacco use. A temporary 6% drop in cigarette consumption occurred in 1954 [1], before a rise, and then another drop following the 1964 Surgeon General's Report [33]. Further evidence was provided by a 1965 study conducted in New York State, where Hammond and colleagues [34] reported higher death rates in cigarette smokers of the general population when compared to non-smokers. Among US men born between 1870 and 1929, the percentage of current smokers was shown to decrease from 48% in 1959, to 38% in 1965 [35]. The smoking behaviour of American physicians during this period appears to have preceded societal trends somewhat, with it having been reported as early as 1962 [36], that cigarette smoking among physicians had "substantially" declined. Not all comments in the US medical literature supported this changing tide of opinion however, with one article in 1959[37] for example, commenting that "..we might as well continue to smoke and enjoy ourselves" (p.60). Regarding the need to quit smoking, another editorial in 1964 [38] stated that physicians should "try it *once!*" (italics from original text, p.408). Furthermore, if physicians then failed in their own quitting attempts, they should then "forget about it" (p.408) [38].

### Smoking Rates in the US and Other Countries

Some of the earliest research to look at smoking among US physicians appears to have been published by Vaillant and colleagues [20], who conducted a 20-year longitudinal study of "mood-altering drugs", including tobacco. Their investigation began in the 1930s, and reported smoking rates among the same group of 45 physicians between 1949 and 1967. Tobacco use has also been well-studied longitudinally within the British Doctor's Study [39-44]. Despite this fact, relatively few studies of physician's smoking habits appear to have been conducted in the late 1940 early 1950s, notwithstanding Doll and Hill's [40] report, where 87% of British physicians were smoking in 1951. Vaillant et al's[20] group had reduced their smoking prevalence from 46% in 1953 to 43% in 1957, while Snegireff and Lombard [45] reported a prevalence rate of 35% in 1954. By the 1960s, smoking research was becoming more common among physicians, with at least 27 investigations being conducted in the US. In other parts of the world, 39% of New Zealand physicians were reported to be current smokers in 1963 [46], while a 1964 study from Australia [47] found that 27% of physicians smoked cigarettes, 14% pipes and 10% cigars. Nishizumi and Kuratsune [48] revealed that 68% of male and 19% of female Japanese physicians smoked in 1965, a rate that was 49% and 27% respectively among their Irish counterparts during 1967 [49]. Although the year of study was not clearly defined, in 1968 Phillips and Taylor [50] published a study describing a cigarette smoking rate of 35% among Canadian physicians. Approximately 24% of male and 17% of female physicians were regular smokers in Finland during 1969, with a further 10% and 9% respectively, being irregular smokers [51].

Thirty seven percent of Swedish physicians were smoking in 1972 [52], with 35% and 15% of their male and female counterparts from New Zealand also doing so in the same year [46]. It was reported that 14% of Australian physicians smoked cigarettes and 10% smoked cigars or pipes in 1974 [53], while in Norway, the smoking rates for male and female physicians were 35% and 22% respectively in the same year [54]. Between 1974 and 1977 in the US, Nelson and colleagues [55] reported that 19% of physicians smoked cigarettes, a rate which was very similar to that observed in New Zealand during 1976 (20% for males and 17% for females) [56]. By the late 1970s, 14% of Massachusetts [57] and 13% of Rhode Island [58] physicians were still smoking, although a much higher rate (50%) was reported in 1979 among their Malaysian counterparts [59]. In 1980, smoking rates among physicians in the Sudan was reported to be 46% among males [60], while in 1981, 55% of Dutch [61] and 15% of New Zealand [62] physicians smoked. By 1982, 20% of their Canadian [63] and 40% of their Indian [64] counterparts were using tobacco products. In the US, Stellman and colleagues [65] reported that 15% of male and 21% of female physicians were smoking cigarettes in 1982, while in Rhode Island [66] the rate was 8% in 1983. Other international research conducted in the same year (1983), reported physicians smoking rates of 19% in Scotland [67], 32% in Belgium [68], and 42% in Japan [69].

### Smoking by Medical Specialty

Not all studies on US physicians' smoking habits examined their data by medical speciality. Of those that did, a few interesting and inconsistent trends were observed. In Massachusetts during 1954 for example, Snegireff & Lombard [70] reported that the lowest rate of smoking was among physicians practicing in the field of preventive medicine or public health (45%), whereas the highest rate was in proctology (82%). In 1964, Tate & Fulghum [71] reported that the smoking rate among Florida physicians was 40% in urology, 37% in obstetrics/gynaecology, psychiatry and general practice. In a 1967 study, Coe & Brehm [72] revealed that 31% of internists and 29% of general practitioners smoked. Other early studies by Eisinger [73] and Tamerin & Eisinger [74] revealed that 36% of paediatricians and 42% of psychiatrists, respectively, smoked cigarettes in 1968. In 1972, Fulghum et al [75] reported that the smoking prevalence among Florida obstetricians/gynaecologists was 26%, whereas in general practice it was 20%. Wells et al [76] found that family medicine physicians were the least likely to be smoking in 1978, while Fortmann et al [77] reported that primary care physicians had the highest smoking prevalence rate by speciality, in California during 1982.

Somewhat surprisingly, a study of smoking among *pulmonary *physicians by Sachs [78,79] reported that between 5% and 19% were current smokers, with smoking being more common among non-practicing specialists than practicing specialists. A 1973 study of Rhode Island physicians on the other hand [80], reported no smokers at all within this particular speciality. An investigation of cardiology conference delegates conducted by Stamler in 1984 [81] reported that only 7% were current cigarette smokers. Although the most frequent research on tobacco smoking rates appears to have been conducted in Rhode Island [58,66,82-85], tobacco use among the study participants when considered by medical speciality, were far from uniform. Refer to Table S1 (Additional file 1). This phenomenon makes it difficult to accurately ascertain which subsections of the US medical profession, if any, consistently smoked more frequently than others.

### Other Influences on Smoking Behaviour

A physicians' spouse may be an important influence on whether he or she actually smokes, and women's medical auxiliaries in New York were becoming increasingly active in smoking-related matters by the 1970s [86]. A physician's smoking habit probably reflects that of their partner due to assortative mating [16]. Greenwald et al's [87] 1968 study from New York for example, revealed that physicians who had never smoked tended to have never-smoking wives, and vice versa. Similar trends have also been demonstrated among physicians in New Zealand [88] and Scotland [67]. In the early to mid 20^th ^century it was further demonstrated that US physicians tended to marry other physicians. Three studies of medical school graduates for example [89-91], found that around half of the female physicians also had physician husbands. The influence of a doctor's spouse therefore, may represent a hitherto underexploited "window" for reaching smoking members of the profession with appropriate tobacco control programs.

### Longitudinal Trends Elsewhere in the World

From a purely longitudinal perspective, this review has clearly demonstrated a consistent decline in the smoking habits of US physicians occurring since the mid 1950s. Similar trends have also been demonstrated in other countries. Perhaps the most famous of these, the British Doctors' Study, began in 1951 [40], and followed up its cohort after four [92], ten [41], twenty [42,93], forty [44] and fifty [43] years. Among Doll et al's group, the proportion of smokers declined from 62% in 1951 to 18% by 1990 [44]. Aside from the United Kingdom [43], other studies have also revealed longitudinal smoking trends among physicians in Scandinavia [94] and Holland [95]. In a summary of previous research, van Reek and Adriaanse [94] reported that smoking among male physicians declined from 74% (1952) to 19% (1984) in Norway, from 34% (1969) to 19% (1984) in Finland, and from 64% (1970) to 28% (1989) in Denmark. In Sweden, Faith-Ell and Wilhelmsen [52] reported that the national smoking rate of physicians declined from 46% in 1969 to 37% in 1972. Aside from current smokers, Adriaanse and colleagues [95] reported that the proportion of never-smoking physicians in the Netherlands increased from 16% in 1977–78, to 18% in 1981 and then to 27% in 1983. Between 1966 and 1967 in the United Kingdom, Fletcher and Doll [96] revealed a continuing trend for more smoking doctors to quit, than for ex-smokers to start again. Early smoking trends were also well-documented among physicians in New Zealand for many years [46,56,62,88,97], as it was one of the first countries to include smoking questions on its national census [97]. Summarising these investigations, Hay [97] reported that the smoking rate of New Zealand physicians declined from 20% in 1976, to 15% in 1981, and finally, to 5% in 1996. In the US, longitudinal smoking trends among physicians appear to have been the most well-studied in Rhode Island, where it was shown to decline 73% between 1963 and 1983 [98].

### Quitting Smoking

Encouraging physicians who currently smoke to quit their habit remains a contentious issue for tobacco control. On one hand, the British Doctor's Study revealed that quitting smoking at any age is clearly effective at reducing the loss of life expectancy due to smoking [99]. On the other, as this review has shown, the US medical profession was still not entirely smoke free by 1984, even though physicians were known to have given up smoking at a higher rate than any other professional group [100]. Targeting medical students may represent one way forward in this regard, although ingrained smoking habits that begin in medical school may be particularly difficult to address. A previous study of Malaysian doctors for example [101] found that around half were already smoking before they even entered medical school. Some of the earliest US studies [102,103] also revealed that a large proportion of medical students were using tobacco products at that time, although contemporary research suggests that US medical students now have some of the lowest rates of smoking in the world [104], similar to their dental student counterparts [105]. Whether medical students who smoke should be advised not to enter certain medical specializations however, remains another issue for debate [106].

## Conclusion

Overall, it can be clearly seen that 1949 to 1984 was a pivotal era in the decline of tobacco smoking among US physicians. From being an ingrained behaviour in the mid 20^th ^century, tobacco use became steadily less common over time as the medical profession, like the society it served, became increasingly aware of the dangers that smoking incurred for health. Physicians have always had an important responsibility to convince their patients not to smoke, as they are generally viewed as exemplars by the community, and also serve as providers of support, information and encouragement in helping patients to achieve such a goal. Although once used for cigarette advertising, US physicians were largely absent from tobacco-related advertising after the 1950s and the Surgeon General's Report of 1964. As such, it can be assumed that this time period represents the beginning of a major change within American society. By the 1950s US physicians had begun to question the safety of tobacco products, and by the 1960s and 1970s this had resulted in a continuous decline in their use. By the 1980s, as this review has shown, few US physicians were still smoking, and many of their younger counterparts had probably never smoked at all. Nevertheless, the fact that any physicians continued to smoke is still unfortunate, given their undoubted status as role models. Either way, much can be learned from this important era in tobacco control, and as such, it is important that physician's smoking in the US, as elsewhere, continues its decline so that the medical profession can remain at the forefront of tobacco control programs and continue to lead the way as preventive medicine exemplars.

## Competing interests

The author declares that they have no competing interests.

## Supplementary Material

Additional file 1 Results of Tobacco Smoking Surveys Conducted among Physicians in the United States between 1949 and 1984 (Arranged by Geographic Location and Date of Survey) Click here for file

## References

[B1] Garfinkel L (1997). Trends in cigarette smoking in the United States. Preventive medicine.

[B2] Giovino GA (2002). Epidemiology of tobacco use in the United States. Oncogene.

[B3] Mahaney FX (1994). Oldtime ads tout health benefits of smoking: tobacco industry had doctors' help. Journal of the National Cancer Institute.

[B4] Gardner MN, Brandt AM (2006). "The doctors' choice is America's choice": the physician in US cigarette advertisements, 1930–1953. American journal of public health.

[B5] (1983). When "more doctors smoked Camels": cigarette advertising in the Journal. New York state journal of medicine.

[B6] Bartrip P (1998). Pushing the weed: the editorializing and advertising of tobacco in the Lancet and the British Medical Journal, 1880–1958. Clio medica (Amsterdam, Netherlands).

[B7] Kawane H (1993). When doctors advertised cigarettes. Tob Control.

[B8] (1964). Smoking and Health. Report of the advisory committee to the surgeon general of the public health service.

[B9] (2005). The role of health professionals in tobacco control.

[B10] Garfinkel L (1976). Cigarette smoking among physicians and other health professionals, 1959–1972. CA: a cancer journal for clinicians.

[B11] (1993). Physician and other health-care professional counseling of smokers to quit – United States, 1991. Mmwr.

[B12] Nett LM (1990). The physician's role in smoking cessation. A present and future agenda. Chest.

[B13] Garfinkel L, Stellman SD (1986). Cigarette smoking among physicians, dentists, and nurses. CA: a cancer journal for clinicians.

[B14] Davis RM (1993). When doctors smoke. Tob Control.

[B15] (1993). Smoking control among health-care workers – World No-Tobacco Day, 1993. Mmwr.

[B16] Smith DR, Leggat PA (2007). An international review of tobacco smoking in the medical profession: 1974–2004. BMC Public Health.

[B17] Giovino GA, Henningfield JE, Tomar SL, Escobedo LG, Slade J (1995). Epidemiology of tobacco use and dependence. Epidemiologic reviews.

[B18] (1957). Doctors have changed their smoking habits. Medical times.

[B19] Little DM (1971). Physician, heal thyself. Anesthesiology.

[B20] Vaillant GE, Brighton JR, McArthur C (1970). Physicians' use of mood-altering drugs. A 20-year follow-up report. The New England journal of medicine.

[B21] Enstrom JE, Kanim LE (1984). Smoking cessation among California physicians: an example of cancer control. Progress in clinical and biological research.

[B22] Glanz K, Fiel SB, Walker LR, Levy MR (1982). Preventive health behavior of physicians. Journal of medical education.

[B23] Samp RJ (1963). Wisconsin physicians and cigarette smoking. Wisconsin medical journal.

[B24] Smith DR, Leggat PA (2006). A comparison of tobacco smoking among dentists in 15 countries. International dental journal.

[B25] Smith DR, Leggat PA (2007). An international review of tobacco smoking research in the nursing profession, 1976–2006. Journal of Research in Nursing.

[B26] Scott HD, Tierney JT, Buechner JS, Waters WJ (1992). Smoking rates among Rhode Island physicians: achieving a smoke-free society. American journal of preventive medicine.

[B27] Brill AA (1922). Tobacco and the individual. Int J Psychoanal.

[B28] Spain DM (1959). The duty of the physician towards his patients in regard to cigarette smoking. CA: a cancer journal for clinicians.

[B29] (1957). SMOKING and health; joint report of the Study Group on Smoking and Health. Science (New York, NY).

[B30] Hammond EC, Horn D (1954). The relationship between human smoking habits and death rates: a follow-up study of 187,766 men. Journal of the American Medical Association.

[B31] Hammond EC, Horn D (1958). Smoking and death rates: report on forty-four months of follow-up of 187,783 men. 2. Death rates by cause. J Am Med Assoc.

[B32] Hammond EC, Horn D (1958). Smoking and death rates; report on forty-four months of follow-up of 187,783 men. I. Total mortality. Journal of the American Medical Association.

[B33] Blum A, Solberg E, Wolinsky H (2004). The Surgeon General's report on smoking and health 40 years later: still wandering in the desert. Lancet.

[B34] Hammond EC, Van Griethuysen TH, Dibeler JB, Sneddon AM, Halligan W (1965). Smoking habits and disease in New York State. New York state journal of medicine.

[B35] Hammond EC, Garfinkel L (1968). Changes in cigarette smoking 1959–1965. American journal of public health and the nation's health.

[B36] Taylor HC (1962). Physicians and cigarette smoking. JAMA.

[B37] Garland LH (1959). The smoking physician. CA: a cancer journal for clinicians.

[B38] Long PH (1964). Doctor, Can You Stop Smoking?. Medical times.

[B39] Doll R, Gray R, Hafner B, Peto R (1980). Mortality in relation to smoking: 22 years' observations on female British doctors. British medical journal.

[B40] Doll R, Hill AB (1954). The mortality of doctors in relation to their smoking habits; a preliminary report. British medical journal.

[B41] Doll R, Hill AB (1964). Mortality in Relation to Smoking: Ten Years' Observations of British Doctors. British medical journal.

[B42] Doll R, Peto R (1976). Mortality in relation to smoking: 20 years' observations on male British doctors. British medical journal.

[B43] Doll R, Peto R, Boreham J, Sutherland I (2004). Mortality in relation to smoking: 50 years' observations on male British doctors. BMJ (Clinical research ed).

[B44] Doll R, Peto R, Wheatley K, Gray R, Sutherland I (1994). Mortality in relation to smoking: 40 years' observations on male British doctors. BMJ (Clinical research ed).

[B45] Snegireff LS, Lombard OM (1954). Survey of smoking habits of Massachusetts physicians. The New England journal of medicine.

[B46] Christmas BW, Hay DR (1976). The smoking habits of New Zealand doctors: a review after ten years. The New Zealand medical journal.

[B47] (1964). 4,776 answer MM smoking survey. Mod Med Aust.

[B48] Nishizumi M, Kuratsune M (1967). A survey of smoking habits of physicians in Western Japan. Jpn J Public Health.

[B49] Bourke GJ, Wilson-Davis K, Thornes D (1972). Smoking habits of the medical profession in the Republic of Ireland. American journal of public health.

[B50] Phillips AJ, Taylor RM (1968). Smoking habits of physicians in Canada. Canadian Medical Association journal.

[B51] Vuori H, Himanen P, Hanninen J, Jarvinen M, Rantanen T (1971). The smoking habits of Finnish physicians. Int J Health Educ.

[B52] Faith-Ell P, Wilhelmsen L (1973). [The smoking habits of Swedish doctors in 1969 and 1972]. Lakartidningen.

[B53] Rankin DW, Gray NJ, Hill DJ, Evans DR (1975). Attitudes and smoking habits of Australian doctors. The Medical journal of Australia.

[B54] Aaro LE, Bjartveit K, Vellar OD, Berglund EL (1977). Smoking habits among Norwegian doctors 1974. Scandinavian journal of social medicine.

[B55] Nelson DE, Giovino GA, Emont SL, Brackbill R, Cameron LL, Peddicord J, Mowery PD (1994). Trends in cigarette smoking among US physicians and nurses. JAMA.

[B56] Hay DR (1980). Cigarette smoking by New Zealand doctors: results from the 1976 population census. The New Zealand medical journal.

[B57] Wyshak G, Lamb GA, Lawrence RS, Curran WJ (1980). A profile of the health-promoting behaviors of physicians and lawyers. The New England journal of medicine.

[B58] Burgess AM, Casey DV, Tierney JT, DePalo P (1980). Cigarette smoking by Rhode Island physicians: a fifteen year update. Percentage of physicians who smoke continues to decrease. Rhode Island medical journal.

[B59] Supramaniam V (1980). Habits and attitudes of Malaysian military doctors towards smoking. The Medical journal of Malaysia.

[B60] Ballal SG (1984). Cigarette smoking and respiratory symptoms among Sudanese doctors. East African medical journal.

[B61] Adriaanse H, Halfens R, Drop MJ, van Reek J (1985). Physicians, smoking, and health in the Netherlands. New York state journal of medicine.

[B62] Hay DR (1984). Intercensal trends in cigarette smoking by New Zealand doctors and nurses. The New Zealand medical journal.

[B63] Senior SL (1982). Study of smoking habits in hospital and attitudes of medical staff towards smoking. Canadian Medical Association journal.

[B64] Sharma TD (1988). Smoking declines in a group of Indian doctors. World Health Forum.

[B65] Stellman SD, Boffetta P, Garfinkel L (1988). Smoking habits of 800,000 American men and women in relation to their occupations. American journal of industrial medicine.

[B66] Buechner JS, Perry DK, Scott HD, Freedman BE, Tierney JT, Waters WJ (1986). Cigarette smoking behavior among Rhode Island physicians, 1963–83. American journal of public health.

[B67] Seiler ER (1983). Smoking habits of doctors and their spouses in south east Scotland. The Journal of the Royal College of General Practitioners.

[B68] Joossens L, Demedts M, Prignot J, Bartsch P, Gyselen A (1987). Smoking habits of Belgian physicians: effects of consonancy behaviour and of age. Acta clinica Belgica.

[B69] Kaetsu A, Fukushima T, Moriyama M, Shigematsu T (2002). Smoking behavior and related lifestyle variables among physicians in Fukuoka, Japan: a cross sectional study. Journal of epidemiology/Japan Epidemiological Association.

[B70] Snegireff LS, Lombard OM (1955). Comparative study of smoking habits of physicians. The New England journal of medicine.

[B71] Tate CI, Fulghum JE (1965). Seventy Per Cent of Florida Physicians Are Nonsmokers. The Journal of the Florida Medical Association.

[B72] Coe RM, Brehm HP (1971). Smoking habits of physicians and preventive care practices. HSMHA health reports.

[B73] Eisinger RA (1972). Cigarette smoking and the pediatrician. Findings based on a national survey. Clinical pediatrics.

[B74] Tamerin JS, Eisinger RA (1972). Cigarette smoking and the psychiatrist. The American journal of psychiatry.

[B75] Fulghum JE, Groover ME, Williams AC, Braatz W (1972). Smoking habits of Florida physicians revisited. JFMA, the Journal of the Florida Medical Association.

[B76] Wells KB, Lewis CE, Leake B, Ware JE (1984). Do physicians preach what they practice? A study of physicians' health habits and counseling practices. JAMA.

[B77] Fortmann SP, Sallis JF, Magnus PM, Farquhar JW (1985). Attitudes and practices of physicians regarding hypertension and smoking: The Stanford Five City Project. Preventive medicine.

[B78] Sachs DP (1983). Smoking habits of pulmonary physicians. The New England journal of medicine.

[B79] Sachs DP (1984). Treatment of cigarette dependency. What American pulmonary physicians do. The American review of respiratory disease.

[B80] Challberg K (1979). Smoking habits of Rhode Island physicians. Rhode Island medical journal.

[B81] Marwick C (1984). Many physicians following own advice about not smoking. JAMA.

[B82] Burgess AM, Casey DB, Tierney JT (1978). Cigarette smoking by Rhode Island physicians, 1963–1973: comparison with lawyers and other adult males. American journal of public health.

[B83] Burgess AM, Tierney JT (1969). Rhode Island physicians' smoking habits revisited 1963–1968. Rhode Island medical journal.

[B84] Burgess AM, Tierney JT (1970). Bias due to nonresponse in a mail survey of Rhode Island physicians' smoking habits – 1968. The New England journal of medicine.

[B85] Murphy TH, Tierney JT (1963). Current Status of Cigarette Smoking among Rhode Island Physicians. Rhode Island medical journal.

[B86] Payne VB (1974). To smoke or not to smoke. New York state journal of medicine.

[B87] Greenwald P, Nelson D, Greene D (1971). Smoking habits of physicians and their wives. New York state journal of medicine.

[B88] Hay DR, Christmas BW (1976). The smoking habits of women doctors and doctors' wives in New Zealand. Preventive medicine.

[B89] Dykman RA, Stalnaker JM (1957). Survey of women physicians graduating from medical school 1925–1940. Journal of medical education.

[B90] Powers L, Parmelle RD, Wiesenfelder H (1969). Practice patterns of women and men physicians. Journal of medical education.

[B91] Shapiro CS, Stibler BJ, Zelkovic AA, Mausner JS (1968). Careers of women physicians: a survey of women graduates from seven medical schools, 1945–1951. Journal of medical education.

[B92] Doll R, Hill AB (1956). Lung cancer and other causes of death in relation to smoking; a second report on the mortality of British doctors. British medical journal.

[B93] Doll R, Peto R (1978). Cigarette smoking and bronchial carcinoma: dose and time relationships among regular smokers and lifelong non-smokers. J Epidemiol Community Health.

[B94] van Reek J, Adriaanse H (1991). Smoking by physicians in Scandinavia: 1952–1989. Scandinavian journal of social medicine.

[B95] Adriaanse H, van Reek J, Metsemakers J (1986). Smoking behaviour of Dutch general practitioners in the period 1977–1983. Scandinavian journal of primary health care.

[B96] Fletcher C, Doll R (1969). A survey of doctors' attitudes to smoking. British journal of preventive & social medicine.

[B97] Hay DR (1998). Cigarette smoking by New Zealand doctors and nurses: results from the 1996 population census. The New Zealand medical journal.

[B98] Scott HD, Fulton JP, Buechner JS, Waters WJ, Tierney JT (1990). Current trends: smoking-related mortality decline among physicians – Rhode Island. Mmwr.

[B99] Boyle P (2005). Tobacco smoking and the British doctors' cohort. British journal of cancer.

[B100] Clever LH, Arsham GM (1984). Physicians' own health – some advice for the advisors. The Western journal of medicine.

[B101] Yaacob I, Abdullah ZA (1993). Smoking habits and attitudes among doctors in a Malaysian hospital. Southeast Asian J Trop Med Public Health.

[B102] Mausner JS (1966). Smoking in medical students. A survey of attitudes, information, and smoking habits. Archives of environmental health.

[B103] Thomas CB, Ross DC, Higinbothom CQ (1964). Precursors of Hypertension and Coronary Disease among Healthy Medical Students: Discriminant Function Analysis. I. Using Smoking Habits as the Criterion. Bulletin of the Johns Hopkins Hospital.

[B104] Smith DR, Leggat PA (2007). An international review of tobacco smoking among medical students. Journal of postgraduate medicine.

[B105] Smith DR, Leggat PA (2007). An international review of tobacco smoking among dental students in 19 countries. International dental journal.

[B106] Chapman S (1995). Doctors who smoke. BMJ (Clinical research ed).

