# Supplementary material for: The historical decline of tobacco smoking among United States physicians: 1949–1984
Source: Tob Induc Dis. 2008 Sep 9;4(1):9. doi: 10.1186/1617-9625-4-9 (PMC2556033; doi:10.1186/1617-9625-4-9)
Supplement: Additional file 1 — Results of Tobacco Smoking Surveys Conducted among Physicians in the United States between 1949 and 1984 (Arranged by Geographic Location and Date of Survey) [file 1617-9625-4-9-S1.pdf]

Table 1 Results of Tobacco Smoking Surveys Conducted among Physicians in the United States between 1949 and 1984 (Arranged by Geographic Location and Date of Survey)

Details of the Study [a]			Participants [d]		Current Smoker [e]			History [f]		Research Methodology [g]			Miscellaneous [h]
**Authors (Publication Year) [a]**	**Year [b]**	**Location [c]**	**Source**	**Specialty**	**Cigarette**	**Pipe**	**Cigar**	**Former**	**Never**	**Distribution**	**Sample**	**Response**	**Additional Findings / Smoking Rate by Specialty**
**Single States**													
Linn et al (1986)[1]	1984	California	University / Hospital	Mixed	9%	-	-	-	91%	Postal†	211	67%	Of the total group, 4% were daily smokers, 1% weekly smokers and 4% monthly smokers
Fortmann et al (1985)[2]	1982	California	Medical Society	Mixed	8%¶			-	-	Postal†	221	62%	Physicians >46 years had the highest smoking rate. By specialty: Primary Care (13%), Specialists (6%)
Wells et al (1984)[3]	1978	California	Medical Society	Mixed	15%	-	-	-	-	Postal†	151	76%	Family Medicine (14%), Internal Medicine (14%), Surgery / Obstetrics (17%)
CMA (1968)[4]	1966	California	Not Specified	Mixed	21%	-	-	38%	40%	Postal	2921	67%	Physicians aged 55-59 years had the highest smoking rate of all those surveyed (24%)
Thomas (1968)[5]	1968*	Connecticut	County Physicians	Mixed	17%	-	-	55%	19%	Postal	743	73%	Of the county physicians who had smoked in the past, 18% had tried to quit but failed
Thomas (1968)[5]	1968*	Connecticut	Medical Centre	Mixed	25%	-	-	42%	13%	Postal	134	n/s	Of the medical centre physicians who had smoked in the past, 25% had tried to quit but failed
Fulghum et al (1972)[6]	1972	Florida	Not Specified	Mixed	18%	-	-	45%	37%	Postal	5736	66%	Obstetrics / Gynaecology (26%), Urology (25%), Psychiatry (22%), General Practice (20%)
Tate & Fulghum (1965)[7]	1964	Florida	Not Specified	Mixed	30%	-	-	45%	25%	Postal	3467	60%	Urology (40%), Obstetrics / Gynaecology (37%), Psychiatry (37%), General Practice (37%)
Levitt & DeWitt (1970)[8]	1969	Indiana	Medical Association	Mixed	17%	-	-	-	-	Postal	2760	71%	Average smoking rate was 22 cigarettes per day, and 38% of smokers had reduced their consumption
Wyshak et al (1980)[9]	1979	Massachusetts	State Physicians	Mixed	14%	-	-	-	-	Postal	289	70%	Fewer physicians smoked when compared to lawyers in the same study
Monson (1970)[10]	1968	Massachusetts	Medical Society	Mixed	24%(M)	-	-	-	38%	Postal	1080	77%	Over 50% of physicians aged 35 years and older were current smokers
Snegireff & Lombard (1959)[11]	1959	Massachusetts	Medical Society	Mixed	39%	-	-	-	-	Postal	4574	n/s	A further 17% of physicians smoked tobacco of some type other than cigarettes
Snegireff & Lombard (1955)[12]	1954	Massachusetts	Medical Society	Mixed	52%‡	-	-	16-22%	13-17%	Postal	4171	n/s	Lowest rate: Preventive Medicine / Public Health (46%), Highest rate: Proctology (82%)
Snegireff & Lombard (1954)[13]	1954	Massachusetts	Medical Society	Mixed	35%	20%	20%	17%	16%	Postal	4104	n/s	The prevalence of cigarette smoking by gender was 34% for males and 43% for females
Greenwald et al (1971)[14]	1968	New York	Woman's Auxiliary	Mixed	24%¶			~43%‡	~31%‡	Postal†	4260#	81%	Physicians who had never smoked tended to have wives who had also never smoked, and vice versa
Browning & Thorp (1969)[15]	1968	Ohio	Thoracic Society	PUL	10%	-	-	46%	44%	Postal	144	77%	Of the current smokers, 53% had never tried to quit and 47% had previously tried to quit but failed
Weitman et al (1967)[16]	1964	Oregon	Medical Association	Mixed	39%¶			27%	34%	Postal†	1790	90%	Only 13% of dermatologists had never smoked compared to 48% of public health physicians
Meighan & Weitman (1965)[17]	1964	Oregon	Medical Association	Mixed	24%	15%≈		27%‡	34%‡	Postal†	1794	90%	Average number of cigarettes smoked per day by current smokers was 21
Glanz et al (1982)[18]	1980	Pennsylvania	University Staff	Mixed	19%	-	-	19%	-	Personal	296	47%	Other health-related behaviours were also studied, with 61% being frequent alcohol consumers
Boucot & Mausner (1964)[19]	1964	Pennsylvania	Medical Society	Mixed	42%¶			37%	21%	Postal	2489	70%	Of the male smokers, 30% smoked cigarettes only, 52% smoked pipes and / or cigars
Buechner et al (1986)[20]	1983	Rhode Island	Licensed Physicians	Mixed	8%	-	-	-	60%	Postal	1837	82%	Cigarette smoking ranged from 4% in otolaryngology to 14% in obstetrics / gynaecology
Burgess et al (1980)[21]	1978	Rhode Island	Active Practitioners	Mixed	13%	-	-	-	-	Postal	1399	84%	Cigarette smoking rates: 9% (internal medicine) to 19% (otolaryngology or obstetrics / gynaecology)
Burgess et al (1978)[22]	1973	Rhode Island	Licensed Physicians	Mixed	19%	-	-	35%	46%	Postal	1234	89%	Cigarette smoking ranged from 9% in internal medicine, to 28% in obstetrics / gynaecology
Burgess & Tierney (1970)[23]	1968	Rhode Island	Licensed Physicians	Mixed	26%	-	-	-	-	Postal	1026	87%	Major non-response bias in smoking prevalence rates (23%: respondents, 46%: non-respondents)
Burgess & Tierney (1969)[24]	1968	Rhode Island	Licensed Physicians	Mixed	23%	18%≈		29%	30%	Postal	1026	87%	Cigarette smoking rates: 13% (ophthalmology) to 67% ("miscellaneous" physicians)
Murphy & Tierney (1963)[25]	1963	Rhode Island	Medical Society	Mixed	33%	-	-	36%	31%	Postal	752	70%	Cigarette smoking ranged from 10% (dermatologists) to 58% ("miscellaneous")

Samp (1963)[26]	1963*	Wisconsin	Medical Society	Mixed	39%‡	-	-	13%‡	-	Meetings	652	40%¤	A further 5% of physicians had "cut down considerably" compared to their prior smoking habits
**Multiple States**													
Covey & Wynder (1981)[27, 28]	1977-79	AL, CA, IL, NY, PN	Hospital Patients	PH/DN	11%!!	16%≈!!		70%!!	46%!!	Interview	61!!	n/s	7% of physicians and / or dentists smoked over 21 cigarettes per day
ACS (1981)[29]	1981*	CA, DL, GE, PN, RI, WI	ACS Unit Members	GP, IM, OBG, O	13%	-	-	-	-	Interview	494	n/s	Smoking physicians were much less likely to speak forcefully to patients about quitting smoking
Lipp & Benson (1972)[30]	1971	CA, NB, NY	Medical Association	Mixed	21%	-	-	40%	-	Postal	1314	47-53%	Physicians in Nebraska (29%) were more likely to smoke cigarettes than in upstate New York (16%)
Vaillant et al (1970)[31, 32]	1967	ML, MS, NY, PN	University Alumni	Mixed	30%‡	28%≈‡		-	-	Longitudinal	45	n/s	Pipe or cigar smokers now comprise almost half of all current tobacco smokers
Vaillant et al (1970)[31, 32]	1964	ML, MS, NY, PN	University Alumni	Mixed	33%‡	31%≈‡		-	-	Longitudinal	45	n/s	Around 5% of current cigarette smokers are now consuming 2 or more packs per day
Vaillant et al (1970)[31, 32]	1957	ML, MS, NY, PN	University Alumni	Mixed	43%‡	19%≈‡		-	-	Longitudinal	45	n/s	Similar proportions of current cigarette smokers are consuming 1-10 or 10-20 cigarettes per day
Vaillant et al (1970)[31, 32]	1953	ML, MS, NY, PN	University Alumni	Mixed	46%‡	13%≈‡		-	-	Longitudinal	45	n/s	The majority of current cigarette smokers are consuming 10-20 cigarettes per day
Vaillant et al (1970)[31, 32]	1949	ML, MS, NY, PN	University Alumni	Mixed	64%‡	7%≈‡		-	-	Longitudinal	45	n/s	Pipe or cigar smokers comprised only a very small proportion of all current smokers
**National Surveys**													
CA:ACJC (1985)[33]	1984	Nationwide	Practicing Physicians	Mixed	12%¶			-	-	Telephone	1035	91%	97% of physicians were advising patients not to smoke, although only 89% were advising *all* patients
Stellman et al (1988)[34]	1982	Nationwide	General Population	Mixed	15% (M)<br>21% (F)	10% (M)≈<br>0% (F)≈		43% (M)<br>32% (F)	32% (M)<br>47% (F)	ACS CPS-II	872061°	n/s	Average number of cigarettes smoked per day by current smokers was 23 (males) and 21 (females)
Brackbill et al (1988)[35]	1978-80	Nationwide	General Population	Mixed	17%	-	-	58%	57%	NHIS	65	n/s	47% of males smoked 20-39 cigarettes per day and 9% smoked 40 or more cigarettes per day
MMWR (1977)[36]	1975	Nationwide	Medical Association	Mixed	21%¶			37%	42%	Postal†	~5000	n/s	36% smoked 1-14 cigarettes per day, 34% smoked 15-24 per day and 29% smoked over 25 per day
Nelson et al (1994)[37]	1974-77	Nationwide	General Population	Mixed	19%	-	-	32%	-	NHIS	137	n/s	There were estimated to be between 32 000 and 71 000 physicians in the US who smoked between 1974-77
Bruce et al (1974)[38]	1967-71	Nationwide	Society Members	ANE	60% (M)‡<br>48% (F)‡	-	-	6%‡	-	Postal	6938	59%	74% of current smokers claimed they smoked 20 or less than cigarettes per day
Sterling & Weinkam (1976)[39]	1970	Nationwide	General Population	PH/SG	16% (WM)	-	-	-	-	HIS	141	n/s	50% of current white male smokers smoked less than one pack of cigarettes per day
Eisinger (1972)[40]	1968	Nationwide	Licensed Physicians	PED	36%	-	-	25%	-	NORCS	287	38%§	Of the paediatricians who had tried to quit smoking, only 46% had been successful
Tamerin & Eisinger (1972)[41]	1968	Nationwide	Licensed Physicians	PSY	42%¶			39%	-	NORCS	309	38%§	Of the psychiatrists who had tried to quit smoking, only 39% had been successful
NCSH (1968)[42]	1968	Nationwide	Licensed Physicians	Mixed	29%	-	-	37%	-	NORCS	4912	38%§	Of the 82% of physicians who had ever smoked, 68% had been successful in quitting
Coe & Brehm (1971)[43]	1967	Nationwide	Practicing Physicians	GP/IM	31%‡	-	-	35%‡	34%‡	Interview	1591	n/s	Internal medicine physicians (31%) were more likely to smoke than general practitioners (29%)
MMWR (1977)[36]	1967	Nationwide	Medical Association	Mixed	30%¶			36%	35%	Postal†	n/s	n/s	31% smoked 1-14 cigarettes per day, 30% smoked 15-24 per day and 39% smoked over 25 per day
**Journal-Based Surveys**													
Patient Care (1976)[44]	1976*	Nationwide	Journal Subscribers	Mixed	27%¶			-	-	Journal	562	56%	62% of all current smokers smoked cigarettes and 37% smoked pipes or cigars
Modern Medicine (1966)[45]	1966	Nationwide	Journal Subscribers	Mixed	23%	-	-	15%‡	45%	Journal	2178	n/s	Over half (55%) reported smoking tobacco products of some description within the past five years
Medical Tribune (1965)[46]	1965*	Nationwide	Journal Subscribers	Mixed	-	10%	11%	38%	27%	Journal	1440∅	n/s	The percentage of ex-smokers rose with age, from 34% (<40 years old), to 42% (>65 years old)
Modern Medicine (1964)[47]	1964	Nationwide	Journal Subscribers	Mixed	23%	19%	15%	-	20%	Journal	56004	n/s	The highest proportion of non-smokers (57%) was among physicians aged over 60 years
Medical Times (1957)[48]	1952-57	Nationwide	Journal Subscribers	GP	-	-	-	-	-	Journal	594	28%	Of the GPs who smoked in 1952, by 1957 17% had quit and 14% had reduced their consumption
Medical Times (1957)[48]	1952-57	Nationwide	Journal Subscribers	RES	-	-	-	-	-	Journal	675	27%	Of the residents who smoked in 1952, by 1957 20% had quit and 19% had reduced their consumption
**Other / Unspecified Locations**													
Marwick (1984)[49]	1984	Not Specified	Conference Delegates	CAR	7%	-	-	-	-	Conference	~500	n/s	Although reported by Marwick,[49] the study was actually conducted by Jeremiah Stamler

Sachs (1983,1984)[50, 51]	1983	Not Specified	Conference Delegates	PUL	5-19%	0-7%≈		-	-	Conference	594	27%	Smoking was higher among non-practicing specialists (19%) than practicing specialists (12%)
Westling-Wikstrand (1970)[52]	1968	Not Specified	University Alumni	Mixed	36% (F)	-	-	14%	42%	Postal	81	n/s	Rate: Professors (59%), Not board-certified (37%), Board certified (14%), Not in practice (25%)
McIlreath & Cohen (1966)[53]	1964	Not Specified	Conference Delegates	Mixed	16%‡	5%‡	4%‡	-	-	Conference	262	n/s	A significantly greater number of cigarette smokers displayed abnormal lung-function test results

Table 1 Legend

[a]Author of study, year of publication and reference number used in this manuscript (ACS=American Cancer Society, CA:ACJC=AC: A Cancer Journal for Clinicians, CMA=California Medical Association, MMWR=Morbidity and Mortality Weekly Report, NCSH=National Clearinghouse for Smoking and Health), [b]Year in which the study was conducted (in cases where the study year was not listed, the publication year is listed and marked with an asterisk*), [c]State in which the study was undertaken (AL=Alabama, CA=California, DL=Delaware, IL=Illinois, MS=Massachusetts, ML=Maryland, NB=Nebraska, NY=New York, PN=Pennsylvania, RI=Rhode Island, WI=Wisconsin), [d]From where the participants were sourced (using terms as described in the manuscript) and whether their medical specialty was listed (CAR=Cardiologists, GP=General Practitioners, IM=Internal Medicine, O=Other or undefined speciality, OBG=Obstetrics / Gynaecology, PED=Paediatricians, PH/DN=Physicians/Dentists, PH/SG=Physicians/Surgeons, PUL=Pulmonary Physicians, PSY=Psychiatrists, RES=Medical Residents), [e]Smoking status as defined in the manuscript and categorized (when available) with all smoking rates rounded to the nearest whole number (M=Male, F=Female, WM=White Male), [f] Prior history of smoking, ¶The type of tobacco smoked was not defined, ≈Current smoker of pipes or cigars or both, ‡Rates calculated by the author, !!The smoking rates of physicians and dentists were combined, [g]Methodology used in the study with response rates rounded to the nearest whole number, †Other methods were used to follow-up initial non-responders (mainly telephone calls), #Response rate of the physician's wives (physicians' smoking habits were reported by their wives), ¤Response rate calculated by the author, °Total participants in the study (not only physicians), ∅Response rate stated as "nearly", §For physician data extracted from national surveys, the overall response rate of the entire survey is listed (ACS=American Cancer Society, HIS=Health Interview Survey, NHIS=National Health Interview Survey, NORCS=National Opinion Research Center Survey), n/s=the response rate of the survey was not specified, [h]Additional information from the study (including smoking rates by medical speciality, where available)

Table 1 References

1. Linn LS, Yager J, Cope D, Leake B: **Health habits and coping behaviors among practicing physicians**. *The Western journal of medicine* 1986, **144**(4):484-489.

2. Fortmann SP, Sallis JF, Magnus PM, Farquhar JW: **Attitudes and practices of physicians regarding hypertension and smoking: The Stanford Five City Project**. *Preventive medicine* 1985, **14**(1):70-80.

3. Wells KB, Lewis CE, Leake B, Ware JE, Jr.: **Do physicians preach what they practice? A study of physicians' health habits and counseling practices**. *JAMA* 1984, **252**(20):2846-2848.

4. **The smoking study. A report of the attitudes and habits of California physicinas with respect to cigarette smoking**. *California medicine* 1968, **109**(4):339-344.

5. Thomas N: **Smoking attitudes of New Haven county physicians: a survey**. *Connecticut medicine* 1968, **32**(12):902-905.

6. Fulghum JE, Groover ME, Jr., Williams AC, Braatz W: **Smoking habits of Florida physicians revisited**. *JFMA, the Journal of the Florida Medical Association* 1972, **59**(10):23-28.

7. Tate CI, Fulghum JE: **Seventy Per Cent of Florida Physicians Are Nonsmokers**. *The Journal of the Florida Medical Association* 1965, **52**:47-48.

8. Levitt EE, DeWitt KN: **A survey of smoking behavior and attitudes of Indiana physicians**. *The Journal of the Indiana State Medical Association* 1970, **63**(4):336-339.

9. Wyshak G, Lamb GA, Lawrence RS, Curran WJ: **A profile of the health-promoting behaviors of physicians and lawyers**. *The New England journal of medicine* 1980, **303**(2):104-107.

10. Monson RR: **Cigarette smoking by Massachusetts physicians--1968**. *The New England journal of medicine* 1970, **282**(16):906-908.

11. Snegireff LS, Lombard OM: **Smoking habits of Massachusetts physicians; five-year follow-up study (1954-1959)**. *The New England journal of medicine* 1959, **261**:603-604.

12. Snegireff LS, Lombard OM: **Comparative study of smoking habits of physicians**. *The New England journal of medicine* 1955, **252**(17):691-696.

13. Snegireff LS, Lombard OM: **Survey of smoking habits of Massachusetts physicians**. *The New England journal of medicine* 1954, **250**(24):1042-1045.

14. Greenwald P, Nelson D, Greene D: **Smoking habits of physicians and their wives**. *New York state journal of medicine* 1971, **71**(17):2096-2098.

15. Browning RH, Thorp D: **Cigarettes. The Ohio Thoracic Society reports**. *The Ohio State medical journal* 1969, **65**(3):245-247.

16. Weitman M, Meighan SS: **Smoking patterns and specialty training of Oregon physicians**. *Cancer* 1967, **20**(6):974-982.

17. Meighan SS, Weitman M: **Smoking: habits and beliefs of Oregon physicians**. *Journal of the National Cancer Institute* 1965, **35**(5):893-898.

18. Glanz K, Fiel SB, Walker LR, Levy MR: **Preventive health behavior of physicians**. *Journal of medical education* 1982, **57**(8):637-639.

19. Boucot KR, Mausner JS: **Smoking among the members of the Philadelphia County Medical Society.** *Philadelphia Medicine* 1964, **59**:711-712.

20. Buechner JS, Perry DK, Scott HD, Freedman BE, Tierney JT, Waters WJ: **Cigarette smoking behavior among Rhode Island physicians, 1963-83**. *American journal of public health* 1986, **76**(3):285-286.

21. Burgess AM, Jr., Casey DV, Tierney JT, DePalo P: **Cigarette smoking by Rhode Island physicians: a fifteen year update. Percentage of physicians who smoke continues to decrease**. *Rhode Island medical journal* 1980, **63**(9):345-347.

22. Burgess AM, Jr., Casey DB, Tierney JT: **Cigarette smoking by Rhode Island physicians, 1963-1973: comparison with lawyers and other adult males**. *American journal of public health* 1978, **68**(1):63-65.

23. Burgess AM, Jr., Tierney JT: **Bias due to nonresponse in a mail survey of Rhode Island physicians' smoking habits--1968**. *The New England journal of medicine* 1970, **282**(16):908.

24. Burgess AM, Jr., Tierney JT: **Rhode Island physicians' smoking habits revisited 1963-1968**. *Rhode Island medical journal* 1969, **52**(8):437-440.

25. Murphy TH, Tierney JT: **Current Status of Cigarette Smoking among Rhode Island Physicians**. *Rhode Island medical journal* 1963, **46**:655-657.

26. Samp RJ: **Wisconsin physicians and cigarette smoking**. *Wisconsin medical journal* 1963, **62**:229.

27. Covey LS, Wynder EL: **Smoking habits and occupational status**. *J Occup Med* 1981, **23**(8):537-542.

28. Wynder EL, Stellman SD: **Comparative epidemiology of tobacco-related cancers**. *Cancer research* 1977, **37**(12):4608-4622.

29. **The impact of providing physicians with quit-smoking materials for smoking patients**. *CA: a cancer journal for clinicians* 1981, **31**(2):75-78.

30. Lipp MR, Benson SG: **Physician use of marijuana, alcohol, and tobacco**. *The American journal of psychiatry* 1972, **129**(5):612-616.

31. Enstrom JE, Kanim LE: **Smoking cessation among California physicians: an example of cancer control**. *Progress in clinical and biological research* 1984, **156**:255-264.

32. Vaillant GE, Brighton JR, McArthur C: **Physicians' use of mood-altering drugs. A 20-year follow-up report**. *The New England journal of medicine* 1970, **282**(7):365-370.

33. **Survey of physicians' attitudes and practices in early cancer detection**. *CA: a cancer journal for clinicians* 1985, **35**(4):197-213.

34. Stellman SD, Boffetta P, Garfinkel L: **Smoking habits of 800,000 American men and women in relation to their occupations**. *American journal of industrial medicine* 1988, **13**(1):43-58.

35. Brackbill R, Frazier T, Shilling S: **Smoking characteristics of US workers, 1978-1980**. *American journal of industrial medicine* 1988, **13**(1):5-41.

36. **Current trends: Smoking behavior and attitudes of physicians, dentists, nurses, and pharmacists, 1975**. *Mmwr* 1977, **26**:185.

37. Nelson DE, Giovino GA, Emont SL, Brackbill R, Cameron LL, Peddicord J, Mowery PD: **Trends in cigarette smoking among US physicians and nurses**. *JAMA* 1994, **271**(16):1273-1275.

38. Bruce DL, Eide KA, Smith NJ, Seltzer F, Dykes MH: **Characteristics of the American Society of Anesthesiologists' membership, 1967-1971**. *Anesthesiology* 1974, **41**(1):67-70.

39. Sterling TD, Weinkam JJ: **Smoking characteristics by type of employment**. *J Occup Med* 1976, **18**(11):743-754.

40. Eisinger RA: **Cigarette smoking and the pediatrician. Findings based on a national survey**. *Clinical pediatrics* 1972, **11**(11):645-647.

41. Tamerin JS, Eisinger RA: **Cigarette smoking and the psychiatrist**. *The American journal of psychiatry* 1972, **128**(10):1224-1229.

42. **National Clearinghouse for Smoking and Health: Survey conducted by the National Opinion Research Center**. Washington: US Public Health Service; 1968.

43. Coe RM, Brehm HP: **Smoking habits of physicians and preventive care practices**. *HSMHA health reports* 1971, **86**(3):217-221.

44. **Special survey - How doctors take care of themselves.** *Patient Care* 1976, **10**(8):49-59.

45. **1966 survey results: Fewer doctors now smoke; patients often ignore advice.** *Mod Med* 1966, **34**(6):14,18.

46. **Doctor, When did you have your last cigarette?** *Med Tribune* 1965, **6**:21.

47. **60,000 answer MM smoking survey: Half of physicians don't smoke; nearly quarter use cigarettes.** *Mod Med* 1964, **32**(5):18,22,26,30.

48. **Doctors have changed their smoking habits**. *Medical times* 1957, **85**(11):1211-1223.

49. Marwick C: **Many physicians following own advice about not smoking**. *JAMA* 1984, **252**(20):2804.

50. Sachs DP: **Smoking habits of pulmonary physicians**. *The New England journal of medicine* 1983, **309**(13):799.

51. Sachs DP: **Treatment of cigarette dependency. What American pulmonary physicians do**. *The American review of respiratory disease* 1984, **129**(6):1010-1013.

52. Westling-Wikstrand H, Monk MA, Thomas CB: **Some characteristics related to the career status of women physicians.** *Johns Hopkins Medical Journal* 1970, **127**(5):273-286.

53. McIlreath FJ, Cohen BM: **Ventilatory performance of American physicians**. *The American journal of the medical sciences* 1966, **252**(1):1-8.
